# Trust Model of Wireless Sensor Networks and Its Application in Data Fusion

**DOI:** 10.3390/s17040703

**Published:** 2017-03-28

**Authors:** Zhenguo Chen, Liqin Tian, Chuang Lin

**Affiliations:** 1 Hebei Engineering Technology Research Center for IOT Data acquisition & Processing, North China Institute of Science and Technology, East Yanjiao, Beijing 101601, China; tianliqin@ncist.edu.cn; 2 School of Computer Science and Engineering, Northeastern University, Shenyang 110819, China; 3 Department of Computer Science and Technology, Tsinghua University, Beijing 100084, China; chlin@tsinghua.edu.cn

**Keywords:** trust evaluation model, wireless sensor networks, sensor data, data fusion

## Abstract

In order to ensure the reliability and credibility of the data in wireless sensor networks (WSNs), this paper proposes a trust evaluation model and data fusion mechanism based on trust. First of all, it gives the model structure. Then, the calculation rules of trust are given. In the trust evaluation model, comprehensive trust consists of three parts: behavior trust, data trust, and historical trust. Data trust can be calculated by processing the sensor data. Based on the behavior of nodes in sensing and forwarding, the behavior trust is obtained. The initial value of historical trust is set to the maximum and updated with comprehensive trust. Comprehensive trust can be obtained by weighted calculation, and then the model is used to construct the trust list and guide the process of data fusion. Using the trust model, simulation results indicate that energy consumption can be reduced by an average of 15%. The detection rate of abnormal nodes is at least 10% higher than that of the lightweight and dependable trust system (LDTS) model. Therefore, this model has good performance in ensuring the reliability and credibility of the data. Moreover, the energy consumption of transmitting was greatly reduced.

## 1. Introduction

In recent years, research and application of the Internet of Things (IoT) have gained worldwide attention [1,2,3,4,5,6]. Wireless sensor networks (WSNs) have been more widely used as a major technology of the IoT. The scale of the data generated by the application of the WSNs has grown substantially. As such, ensuring the reliability and credibility of the sensor data is a problem that people pay close attention to. Since most applications are in the open environment, it is difficult to define the security boundary, and it cannot be ensured that processes such as sensing, transmission, processing, and others do not produce changes. It is difficult to directly apply the traditional security policies and methods in the large-scale deployment of the environment. In addition, many WSN applications are limited by calculation, storage and energy. Therefore, reducing the energy consumption in the process of data transmission is also an important problem in the application of WSNs.

WSNs are composed of many sensor nodes. These nodes are usually used to perform some specific monitoring tasks. They can obtain the monitoring data in an area, and the data are transmitted to the control center for further analysis. However, the open environment of the WSNs makes nodes easily exposed to a variety of attacks, such as eavesdropping, node compromising, and physical disruption. These attacks are likely to lead to unreliable data. Therefore, it is necessary to develop measures to ensure data reliability and reduce energy consumption. 

The motivation in this work is based on the idea that the trust value of an object can be reflected by the data and behavior of the object. For example, consider the case of an object that is abnormal. It will bring abnormal data or behavior. In fact, any application scenarios that involves data-aware and data forwarding can be supported by such a framework.

The trust model consists of three components: data trust, behavior trust, and historical trust. Real time data, regional data, and historical data are considered in the calculation of data trust. This ensures that the data trust is coherent and reduces the possibility of false positives. Behavior trust is based on the statistical value of abnormal behavior. Historical trust is given initially and then updated according to the comprehensive trust. In the model, some thresholds are set. According to the trust value and the threshold value, we can update each trust value.

Our trust model can be thought of as a simple form of anomaly detection. The basis of detection depends on the degree of trust between perceived objects and decision objects. The computation and storage of trust value are all located on the decision objects. The perceived objects of WSNs are only involved in the data collection and forwarding. Therefore, the reliability of the trust value is guaranteed.

Furthermore, the trust model that is introduced in the process of data fusion can reduce the scale of the fused data and can further reduce energy consumption. Therefore, a simple data fusion strategy based on the trust model is presented in this paper. Using the trust model and the given threshold, we can exclude the abnormal data from the fused data.

Our proposal has the following advantages. Firstly, the realization is simple, and it is easy to deploy in a resource-constrained device. Secondly, the trust value is dynamic and can cope with the changing environment. Thirdly, the perception data plays a key role in the calculation of the trust value. Fourthly, the model depends only on the perceived data and the behavior of the object, and is not limited to the specific perception technology.

The rest of the paper is organized as follows. In Section 2, related works are illustrated. In Section 3, the trust evaluation model is proposed. The calculation method of the trust value is described in detail. In Section 4, the method of data fusion using the trust model is given. In Section 5, the model is evaluated based on OMNeT++ platform. The results and analysis are given. Finally, the paper is summarized, and future research directions are given.

## 2. Related Work

Much research has been conducted on effective methods of detecting abnormal nodes and improving the security, credibility, and reliability of WSNs. In Reference [7], a reputation-based framework for sensor networks (RFSN) used a watchdog mechanism to build a trust rating. Within the framework of RFSN, a beta reputation system for sensor networks (BRSN) that used a Bayesian formulation was employed. Then, data fusion can be performed on these weighted data readings, thereby reducing the impact of untrustworthy nodes. Srinivasan et al. [8] proposed a novel reputation-based scheme called Distributed Reputation based Beacon Trust System (DRBTS) for excluding malicious beacon nodes (BNs) that provide false location information. In DRBTS, every BN monitors its one-hop neighborhood for misbehaving BNs and provides information by maintaining and exchanging a neighbor’s reputation. However, these methods only focus on the security of the anchor. Moreover, they need more computation and energy. Chen et al. created a trust model for the IoT that uses fuzzy sets. They focus mainly on different security challenges, such as detecting malicious attacks [9]. A heuristic approach based on trustworthy architecture for wireless sensor networks (WSNs) is proposed in Reference [10]. It considers the challenges of the system and focuses on the collaborative mechanism for trust evaluation and maintenance. To ensure the credibility of the node and the security of data, Jing et al. studied the problem of trust management in WSNs and gave a detailed introduction to the characteristics, taxonomy, and design of the framework, vulnerability analysis, attack models, and countermeasures [11]. Xiao et al. used Gaussian distribution to study the credibility model of a sensor network [12]. Fan proposed a trust evaluation method based on energy monitoring to solve the problem of trust in WSNs [13]. Li et al. designed a clustering WSN that is both lightweight and low in energy consumption [14]. Zhang et al. gave a WSN model based on multi-layer trust [15]. Zheng et al. investigated the properties of trust, proposed objectives of IoT trust management, and provided a survey on the current literature’s advances towards trustworthy IoT [16]. Duan et al. proposed an energy-aware trust derivation scheme using a game theory approach, which manages overhead while maintaining adequate security of WSNs. It can achieve both the intended security and high efficiency that is suitable for WSN-based IoT networks [17]. Sahoo et al. presented a lightweight dynamic trust model along with a honey bee mating algorithm, which will only prevent malicious nodes from becoming a cluster head. The choice of a lightweight trust model made the clustering method more secure and energy efficient [18]. Zhou et al. studied the safety certification of direct trust evaluation and indirect trust evaluation in vehicle networking. It could accurately validate the vehicle node [19]. Labraoui et al. proposed a Risk-aware Reputation-based Trust (RaRTrust) model for WSNs. This framework uses both reputation and risk to evaluate the trustworthiness of a sensor node [20]. Ramos et al. presented a new comprehensive security estimation scheme for WSNs to ensure the sensor data security [21]. Sicaria et al. presented the main research challenges and the existing solutions in the field of IoT security, identify open issues, and make suggestions for future research [22]. Nguyen et al. discussed the applicability and limitations of existing internet protocol-based Internet security protocols and other security protocols used in WSNs, which are potentially suitable in the context of the IoT [23]. Neisse et al. proposed a model-based security toolkit which is integrated in a management framework for IoT devices and supports specification and efficient evaluation of security policies to enable the protection of user data [24]. Li et al. proposed a block-sparse signal reconstruction algorithm based on the adaptive step. Then, the abnormal node is predicted based on the algorithm at the sink node [25]. Wang et al. proposed a mechanism for detecting abnormal readings based on compressed sensing (CS) and a self-regression model which can distinguish between abnormal data and normal data [26]. In Reference [27], the anomaly detection problem is modeled as a weighted L1 norm minimization problem, and the orthogonal matching pursuit (OMP) algorithm is used to solve the problem. In Reference [28], haze monitoring data were calculated and analyzed. Trust evaluation was used to evaluate the source of haze, and achieved good results.

Through the above analyses, it can be seen that trust evaluation is mainly based on node behavior. As we all know, one important function of the WSNs is to collect sensor data. The reliability of the nodes is directly reflected by the sensor data, but in the previous research, data trust is rarely used to determine the trust evaluation. In this paper, a trust evaluation model is constructed based on node behavior and sensor data. Following that, a data fusion method based on a trust model is presented.

## 3. Trust Evaluation Model

The model is mainly used in the perception layer of WSNs. The perception layer of WSNs can be subdivided into the sensor node, relay node, and sink node. The different types of nodes have different behaviors and data. In this paper, we only give the evaluation to the sensor and relay nodes. The sink node directly communicates with the gateway, so its security is relatively easy to guarantee. In the data transmission phase, the sensor nodes collect data and transmit it to the relay nodes (the cluster head). Relay nodes carry out data fusion and transfer data to the sink. The trust value of the sensor nodes is calculated in the cluster heads. The trust value of the cluster head is calculated in the sink head.

**Figure 1:**
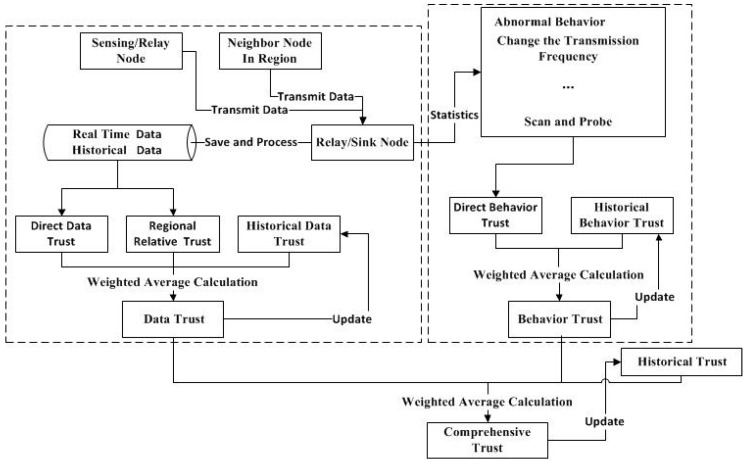
The trust evaluation model.

In this model, the trust evaluation is made up of three parts: behavior trust, data trust, and historical trust. Behavior trust consists of two parts: direct behavior trust and historical behavior trust. Data trust is composed of direct data trust, regional relative trust, and historical data trust. The initial value of historical trust is given and is updated according to the value of comprehensive trust and the threshold value. The trust evaluation model is shown in Figure 1.

### 3.1. Trust Evaluation of Sensor Node

#### 3.1.1. Data Trust of Sensor Node

In order to obtain accurate information, a large number of sensor nodes are usually deployed in the monitoring area. This reduces the precision requirements of individual sensor nodes. In addition, a large number of redundant nodes also give the system strong fault tolerance and can increase the coverage range of the monitoring area and reduce the cave or blind. This feature also gives the opportunity to use monitoring data in conducting trust evaluation. In this paper, data trust is composed of direct data trust, regional relative trust, and historical data trust.

(1) Direct Data Trust

In this model, the relay node is responsible for preserving the historical sensor data. The monitoring indicators are assumed to be continuous, and do not have the characteristics of jump. Since the monitoring indicators are assumed to be continuous, the difference between real-time monitoring data and historical data should theoretically be within a certain range. If the difference between the real-time monitoring data and the historical data is too large, it represents abnormal nodes. So, direct data trust can be calculated by using the real-time monitoring value and the historical data of sensor nodes. The historical data are the average of data in the recent period of time. The real-time monitoring data of the *i*-th sensor node are recorded as ${}^{r}Data_{i}$, and the historical value is recorded as ${}^{h}Data_{i}$. The direct data trust of the *i*-th sensor node is recorded as Tidataddt. The Tidataddt can be calculated by the following equation:

(1) Tidataddt=⌊MAX×(((|rDatai−hDatai|−ddtK)>0?0:||rDatai−hDatai|−ddtK|)/ddtK)⌋

where MAX is a maximum value, and it is set by experience or experts. The value of ${}^{ddt}K$ is the threshold and it is defined as the upper bound of the absolute value of the difference between the real-time monitoring value and the historical value, set by experience or experts. The initial value of Tidataddt is set to MAX and Tidataddt∈[0,MAX].

For example, set ${}^{r}Data_{i}=28.8,\;{}^{h}Data_{i}=26,\;{}^{ddt}K\;=\;5,\;\mathrm{MAX}=100$, then Tidataddt=⌊100×(((|28.7−26|−5)>0?0:||28.7−26|−5|)/5)⌋ = ⌊100×((−2.3>0?0:2.3)/5)⌋ = ⌊100×(2.3/5)⌋=⌊46⌋=46

(2) Regional Relative Trust

Region is a set of sensor nodes that are in communication with the same relay node. Within a certain region, the difference of the real-time data of the same monitoring index should be within a certain range. Based on this, we give the regional relative trust. In order to compute simplicity, we use the weighted average of real-time monitoring values—which comes from the other trusted nodes in the region—to participate in the computation. The regional relative trust of the *i*-th sensor node is calculated by the real-time monitoring data of the *i*-th sensor node and the average value of the real-time monitoring data of other sensor nodes in the region. Assuming that the *i*-th sensor node has n neighbors in the trust list and the data trust of neighbor nodes is greater than or equal to ${\mathrm{Th}}_{\mathrm{susp}}^{\mathrm{data}}$, the average of real-time monitoring data is recorded as ${}^{n}AVER_{i}$, and it can be calculated by Equation (2), wherein j is a natural number.

(2) $${}^{n}AVER_{i}=\sum_{j=1}^{n}{}^{r}Data{}_{j}/n$$

$$dif_{i}^{rrt}=\left\{ \begin{array}{l} {}^{rrt}K,{}^{n}AVER_{i}=0\\ (|{}^{r}Data_{i}-{}^{n}AVER_{i}|-{}^{rrt}K)>0?0:|\left|{}^{r}Data_{i}-{}^{n}AVER_{i}|-{}^{rrt}K\right|,{}^{n}AVER_{i}\neq 0 \end{array}\right.$$

The regional relative trust of the *i*-th sensor node is recorded as Tidatarrt, and it can be calculated by Equation (3):

(3) Tidatarrt=⌈MAX×(difirrt/rrtK)⌉

where MAX is equal to the previous values. The value of ${}^{rrt}K$ is the threshold, and it is defined as the upper bound of the absolute value of the difference between the real-time monitoring value and the average mean of n neighbors, set by experience or experts. The initial value of Tidatarrt is set to MAX and Tidatarrt∈[0,MAX].

(3) Data Trust of Sensor Node

The value of data trust is obtained by the weighted average calculation using direct data trust, regional relative trust, and historical data trust. The direct data trust and regional relative trust can be calculated by Equations (1) and (3), respectively. The initial value of the history data trust is set manually, and then updated according to the data trust value. The data trust of the *i*-th sensor node is recorded as Tidata. The historical data trust of the *i*-th sensor node is recorded as Tidatahdt. The calculation method is shown in Equation (4):

(4) Tidata=⌈α×Tidataddt+β×Tidatarrt+γ×Tidatahdt⌉

where α,β,γ are weighting coefficients, and 0<α,β,γ<1,α+β+γ=1. The user, the experts, and the experience can set its value.

(4) Historical Data Trust

The initial value of historical data trust is set to MAX and updated according to the data trust. The two thresholds are given respectively: suspected abnormal threshold (recorded as ${\mathrm{Th}}_{\mathrm{susp}}^{\mathrm{data}}$) and abnormal threshold (recorded as ${\mathrm{Th}}_{\mathrm{abn}}^{\mathrm{data}}$). The calculation method is shown in Equation (5):

(5) Tidatahdt={Tidata,Tidata<ThsuspdataTidata−|Tidata−Thsuspdata|,Thsuspdata<Tidata<ThabndataTidata−τdata×|Tidata−Thabndata|,Tidata≥Thabndata

In this equation, $\tau_{data}$ ($\tau_{data}\geq 1$) is the penalty coefficient. It can adjust the intensity of punishment.

#### 3.1.2. Behavior Trust of Sensor Node

We judge the state of nodes by collecting the behavior characteristics of nodes. The malicious behaviors of the sensor node mainly include distorted information, injected information, changing of the transmission frequency, scanning, probing, etc. The malicious behavior in tampering with data can be attributed to the data trust evaluation. In this, we only consider changing the transmission frequency, scanning, and probing. Behavior trust is divided into direct behavior trust and historical behavior trust.

(1) Direct Behavior Trust

The transmission frequency of sensor data is usually fixed, and so the transmission frequency becomes one of the important indicators for detecting the node behavior. The standard transmission frequency of the sensor nodes is set to M.The actual transmission frequency of the *i*-th sensor node is denoted as $m_{i}$. When the network is stable, the relay node of each sensor node is stable. The behavior of sensor nodes to scan and detect other relay nodes is usually considered as abnormal. The number of the *i*-th sensor node scanning and probing is recorded as $t_{i}$. The direct behavior trust of the *i*-th sensor node is recorded as Tibehaviordbt. The calculation method is shown in Equation (6):

$$dif_{i}^{dbt}=t_{i}>{\mathrm{TH}}_{\mathrm{sp}}?0:\left|t_{i}-{\mathrm{TH}}_{\mathrm{sp}}\right|$$

(6) Tibehaviordbt=ε1×⌊MAX×((mi<(M−δ)&&mi<(M+δ))?1:0)⌋+ε2×⌊MAX×(difidbt/THsp)⌋

where MAX is equal to the previous values, $\varepsilon_{1}$ and $\varepsilon_{2}$ are weighting coefficients, and $0<\varepsilon_{1},\varepsilon_{2}<1,\varepsilon_{1}+\varepsilon_{2}=1$. The user, experts, and experience can set the value. The value of δ is a fault tolerance factor, and ${\mathrm{TH}}_{\mathrm{sp}}$ is the threshold value, which is used to represent the upper bound of the number of scanning and probing.

(2) Behavior Trust of Sensor Node

The value of behavior trust is obtained by the weighted average calculation, using direct behavior trust and historical behavior trust. The direct behavior trust can be calculated by Equation (6). The initial value of the history behavior trust is set manually and then updated according to behavior trust. The behavior trust of the *i*-th sensor node is recorded as Tibehavior. The historical behavior trust of the *i*-th sensor node is recorded as Tibehaviorhbt. The calculation method is shown in Equation (7):

(7) Tibehavior=⌈λ1×Tibehaviordbt+λ2×Tibehaviorhbt⌉

where $\lambda_{1},\lambda_{2}$ are weighting coefficients, and $0<\lambda_{1},\lambda_{2}<1,\lambda_{1}+\lambda_{2}=1$. The user, experts, and experience can set the value.

(3) Historical Behavior Trust

The initial value of historical behavior trust is set to MAX and updated according to behavior trust. The two thresholds are given, respectively: suspected abnormal threshold (recorded as ${\mathrm{Th}}_{\mathrm{susp}}^{\mathrm{behavior}}$) and abnormal threshold (recorded as ${\mathrm{Th}}_{\mathrm{abn}}^{\mathrm{behavior}}$). The calculation method is shown in Equation (8):

(8) Tibehaviorhbt={Tibehavior,Tibehavior<ThsuspbehaviorTibehavior−|Tibehavior−Thsuspbehavior|,Thsuspbehavior<Tibehavior<ThabnbehaviorTiibehavior−τbehavior×|Tibehavior−Thabnbehavior|,Tibehavior≥Thabnbehavior

In this equation, $\tau_{\mathrm{behavior}}$ ($\tau_{\mathrm{behavior}}\geq 1$) is the penalty coefficient. It can adjust the intensity of punishment.

#### 3.1.3. Comprehensive Trust of Sensor Node

The value of the comprehensive trust is given by the weighted average calculation, using data trust, behavior trust, and historical trust. Data trust and behavior trust can be calculated by Equations (4) and (7), respectively. The initial value of the history trust is set manually and updated according to the comprehensive trust value. The comprehensive trust of the *i*-th sensor node is recorded as $T_{i}$. The historical trust of the *i*-th sensor node is recorded as Tihistory. The calculation method is shown in Equation (9):

(9) Ti=⌈ϕ1×Tidata+ϕ2×Tibehavior+ϕ3×Tihistory⌉

where $\phi_{1},\phi_{2},\phi_{3}$ are weighting coefficients, and $0<\phi_{1},\phi_{2},\phi_{3}<1,\phi_{1}+\phi_{2}+\phi_{3}=1$. The user, experts, and experience can set the value.

#### 3.1.4. Historical Trust of Sensor Node

The historical trust of the sensor nodes is similar to that of the historical data trust and the historical behavior trust. Its initial value is set to MAX. The calculation method is shown in Equation (10):

(10) Tihistory={Ti,Ti<ThsuspTi−|Ti−Thsusp|,Thsusp<Ti<ThabnTi−τ×|Ti−Thabn|,Ti≥Thabn

In this equation, τ (τ≥1) is the penalty coefficient. It can adjust the intensity of punishment. The values of ${\mathrm{Th}}_{\mathrm{susp}}$ and ${\mathrm{Th}}_{\mathrm{abn}}$ are the threshold values.

### 3.2. Trust Evaluation of Relay Node

The relay node is responsible for the trust evaluation of sensor nodes, data forwarding, and data fusion. The sink node executes the trust evaluation of the relay node, and it also includes data trust, behavior trust, and historical trust. The value of the comprehensive trust is also obtained by the weighted calculation of data trust, behavior trust, and historical trust. 

#### 3.2.1. Data Trust

The calculation process of trust in relay nodes is similar to that of sensor nodes. The difference is mainly focused on the data and the definition of region. The data are the fused data in the relay node. The region is a collection of all relay nodes that are limited within a certain range (e.g., a circle with a radius R), or are in communication with the same sink node. Therefore, the calculation process of the relay node is not introduced in detail.

#### 3.2.2. Trust List

**Figure 2:**
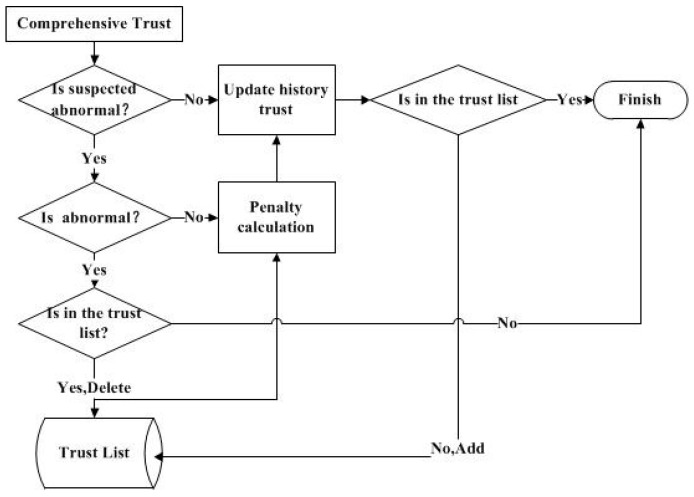
Update of the trust list.

In this paper, a trust list is introduced in order to guarantee the reliability of the data involved in fusion. Each sensor node needs to be authenticated in the access network, so the initial value of the trust list contains all the sensor nodes. In the subsequent operation, the trust list is updated according to the comprehensive trust of each sensor node. The update process is shown in Figure 2.

Figure 2 shows the update process for the trust list and historical trust. According to Equation (10), the following judgment and calculation will be performed. 

If the comprehensive trust is lower than the suspected threshold, then the history trust is equal to the comprehensive trust.

If the comprehensive trust is higher than the suspected threshold, then the anomaly threshold is compared. If it is lower than the anomaly threshold, then the penalty operation is performed to update the historical trust.

If the comprehensive trust is lower than the anomaly threshold, the sensor node is considered to be credible. So, after the historical trust is updated and the trust list is checked, if the sensor node does not exist, it is added to the trust list.

If the comprehensive trust is higher than the anomaly threshold, the penalty factor is introduced to calculate and update historical trust, and then the sensor node is removed from the trust list.

## 4. Trust Evaluation Model Used in Data Fusion

WSNs are often composed of a large number of sensor nodes, so a new scalability challenge is caused by potential collisions and transmissions of redundant data. Regarding energy restrictions, communication should be reduced to increase the lifetime of the sensor nodes. When data fusion is performed (that is, sensor data are fused and only the result is forwarded), the number of messages is reduced, collisions are avoided, and energy is saved. As such, data fusion is one of the important functions of relay nodes.

The existing mechanism of data fusion does not consider the node reliability. For example, items like the median fusion algorithm, the average function fusion algorithm, the heterogeneous fusion algorithm, etc. do not consider the problem of node reliability [29,30,31]. When the data fusion is carried out, the data fusion weights are the same, or the weights are fixed. However, the actual network state is often changed with the dynamic environment. Therefore, this paper proposes a data fusion mechanism based on a trust model. Assuming that the trust list has H nodes, the fusion data are recorded as $data^{f}$. The calculation method is shown in Equation (11).

(11) dataf=∑i=1H(Tidata≥Thsuspdata?Ti×rdatai:0)∑i=1H(Tidata≥Thsuspdata?Ti:0)

**Figure 3:**
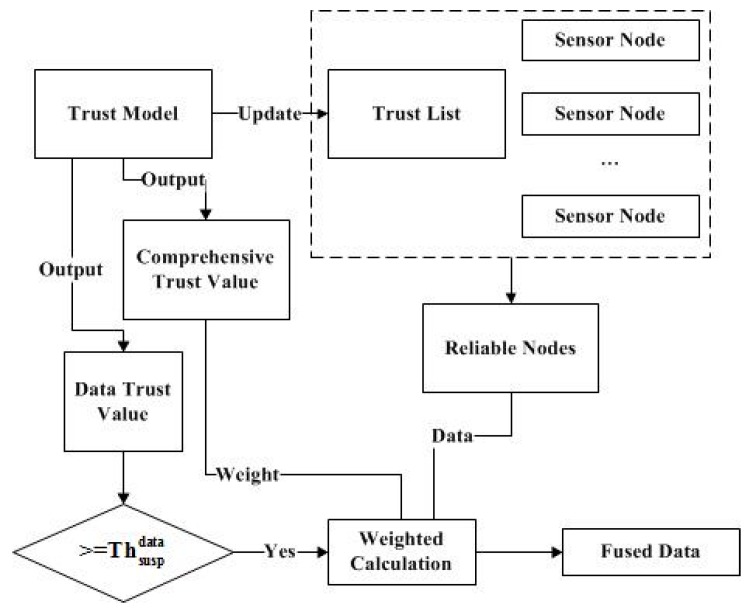
The process of data fusion.

In data fusion, sensor nodes need to satisfy two features. One is that they are included in the trust list. Another has a dynamic weight which changes with the comprehensive trust value. It can make the fusion result more accurate, and can automatically adjust the weight value according to the trust model. The process of data fusion is shown in Figure 3.

## 5. Results and Discussion

The detected data are periodically collected. The cycle is defined as the round, which is set up and broadcast by the sink node. Each round includes a cluster stage and a data transmission phase. In the data transmission phase, the sensor nodes collect data and transmit it to the cluster head (relay nodes). Cluster heads carry out data fusion and transfer data to the sink. The cluster head is dynamic. It is calculated by the sink node in each round. When the node is selected as the cluster head, it is no longer in charge of data sensing. The trust value of the sensor nodes is calculated in the cluster heads. The trust value of the cluster head is calculated in the sink head.

### 5.1. Simulation Environment and Parameter Setting

The experiments are carried out to evaluate the performance of the algorithm on the OMNeT++ platform. In simulations, we have used the same radio model. The solar low-energy adaptive clustering hierarchy (sLeach) protocol is used for clustering [32]. The parameters of the simulation environment are set as shown in Table 1.

In order to calculate the trust value, the trust model introduces several parameters. The parameters of the trust model are shown in Table 2. To simplify the simulation process, $\left({\mathrm{Th}}_{\mathrm{abn}}^{\mathrm{data}}{,\mathrm{Th}}_{\mathrm{susp}}^{\mathrm{data}}\right)$, $\left({\mathrm{Th}}_{\mathrm{abn}}^{\mathrm{behavior}},{\mathrm{Th}}_{\mathrm{susp}}^{\mathrm{behavior}}\right)$, and $\left({\mathrm{Th}}_{\mathrm{abn}}{,\mathrm{Th}}_{\mathrm{susp}}\right)$ have the same value. The values of $\tau_{data}$ and $\tau_{\mathrm{behavior}}$ take a fixed value.

### 5.2. Simulation and Analysis

#### 5.2.1. Node Distribution and Topology

**Figure 4:**
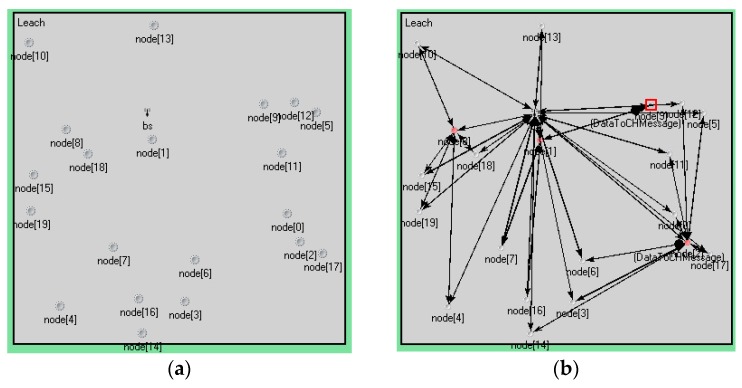

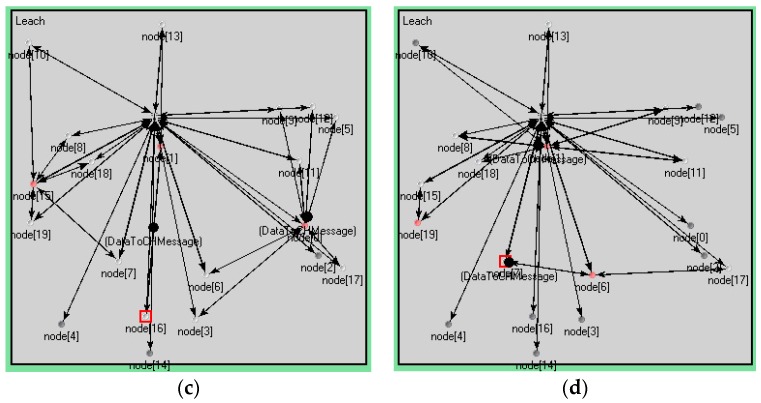
Node distribution and topological graph. (**a**) The graph of node distribution; (**b**) Topological graph of round 1; (**c**) Topological graph of round 2; (**d**) Topological graph of round 3.

In the experiment, the number of nodes was 20. The sink node was set to 1. The cluster head nodes—which are calculated by the sink node—were set to 3. Each experiment had 3 rounds, and each round of the cluster head node was to be selected again. The nodes were randomly distributed in a square area. The detailed values are shown in Table 1. Figure 4 is a graph of node distribution and a topological graph with 20 nodes. Cluster heads and nodes within a cluster in each round are shown in Table 3. Figure 4 and Table 3 show that the cluster heads and topologies of each round were not exactly the same.

#### 5.2.2. Simulation Data

**Figure 5:**
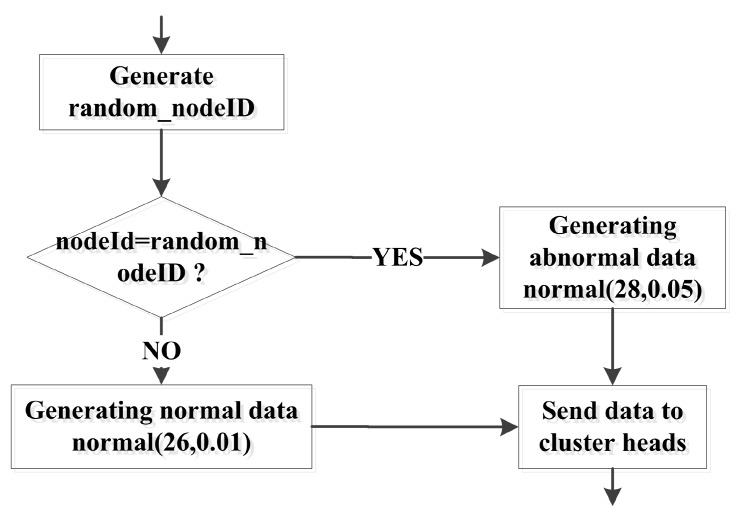
Data generation process.

In the experiment, all the nodes monitor the same index, and the data were obtained by the normal distribution random function in OMNET++. Nodes were randomly selected to generate abnormal data. A data generation algorithm was executed in each node. The data generation process is shown in Figure 5. The norm is a function that was used to calculate the normal random distribution.

#### 5.2.3. Improvements in Energy and Node Survival

The simulation was done on the basis of a sLeach. In this paper, the trust evaluation module was added in the solar leach algorithm. The parameters of the trust model are shown in Table 2.

**Figure 6:**
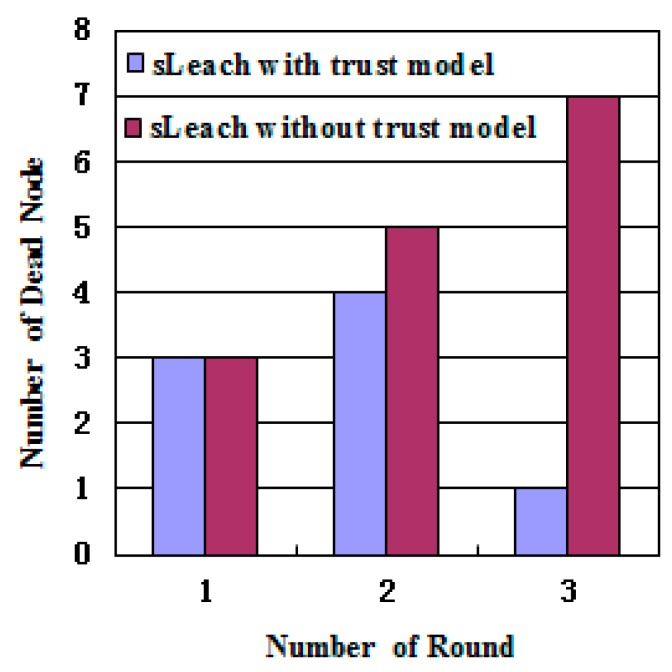
Comparison of death nodes. sLeach: Solar low-energy adaptive clustering hierarchy algorithm.

**Figure 7:**
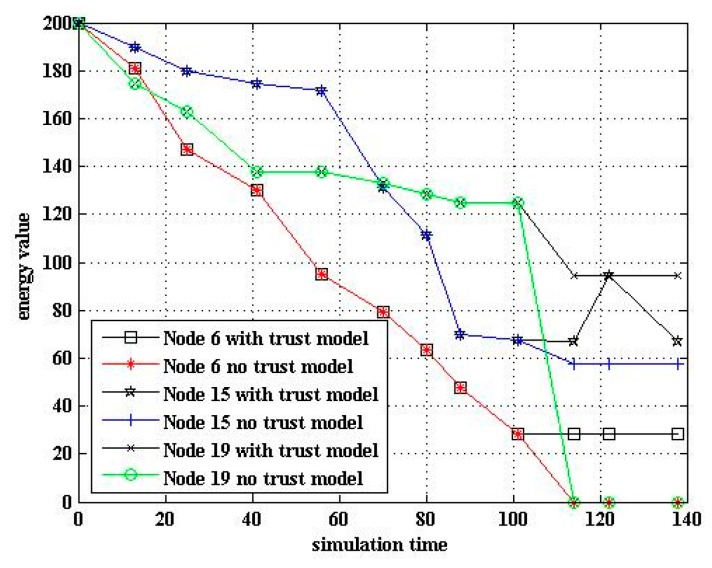
Energy comparison of algorithms.

In WSNs, the main energy consumption comes from communication. In the experiment, the connection between the node and the cluster head was managed through the trust value. When the trust value of the sensor nodes was less than the abnormal threshold, the cluster heads were disconnected, reducing the energy consumption. As can be seen from Figure 6, the algorithm with the trust model had fewer dead nodes than that without it. Therefore, the trust model could improve the survival rate of the nodes. Figure 7 shows the trend of the energy of cluster heads 6, 15, and 19 in two cases of algorithms with and without the trust model. Other nodes had small differences in energy consumption.

#### 5.2.4. Comparison of Trust Value

**Figure 8:**
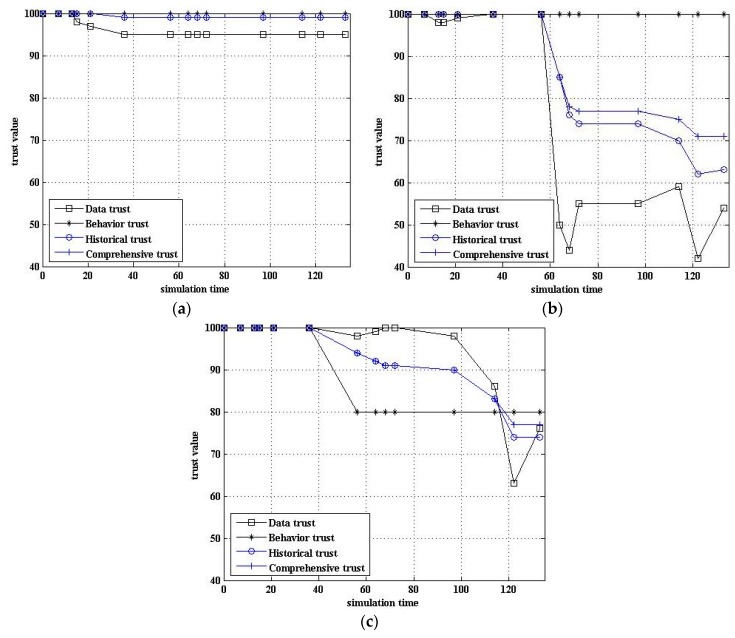
Comparison of trust value in different state. (**a**) Normal node; (**b**) Node with abnormal data; (**c**) Node with suspected data and behavior.

When the node is in a different state, the change trend of the node’s trust value also reflects the validity of the model. Figure 8 shows the change trend of the node trust value in different states.

Figure 8a shows that the trust value of normal nodes was not less than 90. Figure 8b shows that the data trust was dramatically reduced when the sensor data were abnormal. However, due to the inertia, the comprehensive trust was still maintained at a relatively high level. As can be seen, the trust value was gradually restored if the abnormal data were an individual. However, the comprehensive trust was reduced quickly if the abnormal data were present continuously or for long periods. Figure 8c shows that the trust value was reduced if suspected data or suspected behavior was present. In this case, the comprehensive trust was usually greater than the suspected threshold.

#### 5.2.5. Comparison of Fusion Data

**Figure 9:**
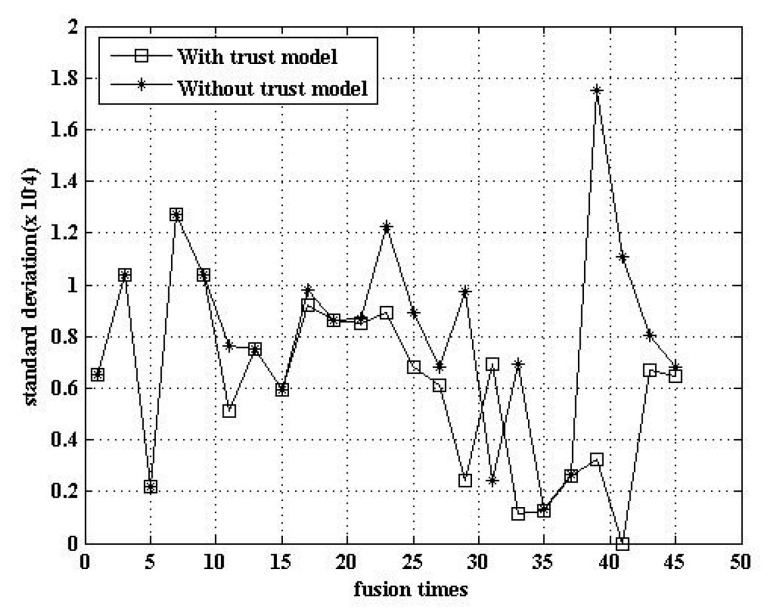
Comparison of fusion data.

The trust model can improve the accuracy of the data fusion. Figure 9 shows that the data fusion using the trust model has a lower standard deviation. That is, the degree of data deviation was reduced after the adoption of the trust model.

#### 5.2.6. Comparison of Abnormal Detection Rate

**Figure 10:**
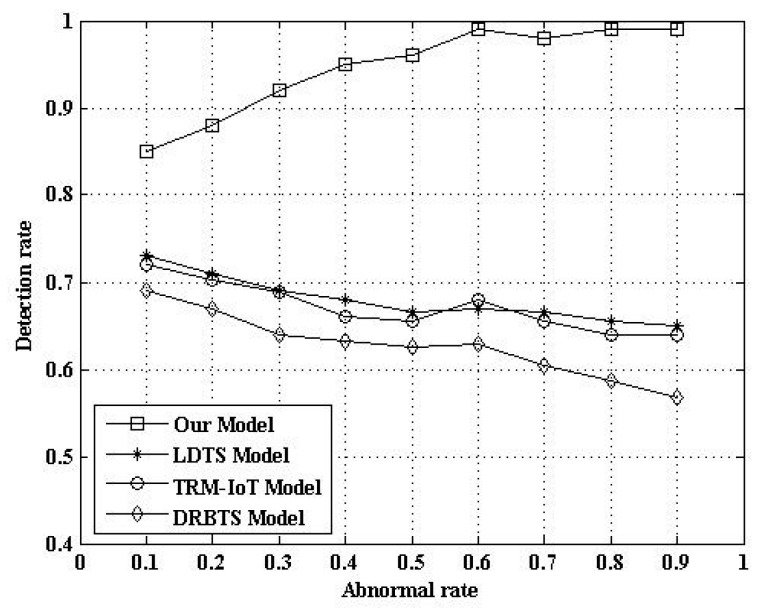
Comparison of abnormal detection rate. DRBTS: distributed reputation-based beacon trust system; LDTS: lightweight and dependable trust system; TRM-IoT: trust management model based on fuzzy reputation for the Internet of Things (IoT).

In order to evaluate the effect of the detection of abnormal nodes, we compared our model with a lightweight and dependable trust system (LDTS) model from the literature [14], the trust management model based on fuzzy reputation for IoT (TRM-IoT), and the distributed reputation-based beacon trust system (DRBTS). The LDTS employs a clustering algorithm and constructs a lightweight trust evaluation scheme and a dependability-enhanced trust evaluation approach. It can adaptively adjust the weights for cluster heads’ (CHs) trust aggregation. This approach can effectively reduce networking consumption while preventing malicious, selfish, and faulty CHs. However, this method only considers data forwarding and communication behavior. It does not consider the role of sensor data. Therefore, the detection ability of abnormal nodes can be further improved. The TRM-IoT is also a trust and reputation model based on behavior. Its advantage is the use of fuzzy theory [9]. The simulation result is shown in Figure 10. Figure 10 indicates that the detection rate of our model is higher than that of the LDTS model. The reason is that our model includes three factors: data, behavior, and historical inertia. The LDTS model only deals with behavioral factors.

## 6. Conclusions

In this paper, we proposed a novel trust evaluation model. Not only can this model make full use of the sensor data, it also includes behavioral data and historical data. It can detect the state of a node according to the data trust. Compared with the evaluation model (which only focuses on the behavior trust), our model has a higher abnormal detection rate. In addition, the trust value is calculated by a simple weighted average method. Therefore, it is light enough to fit well with WSNs without great overheads. At the same time, a trust list can be constructed and updated dynamically by a trust evaluation model. According to the trust list, the data fusion considers only the data of a trusted node, and so the communication cost is saved and the energy consumption is reduced. Simulation results based on OMNET++ show that the trust model can better monitor the status of nodes and can improve the survival time of the node. Furthermore, the trust model has a better anomaly detection rate than the LDTS model. We expect that our trust evaluation model can help to make sensor data more reliable.

The process of effectively improving the fusion of multi-source data and ensuring the reliability of the transmission process will be carried out in future research.

## Figures and Tables

**Table 1:** Parameter values of the simulation environment.

Parameter	Value	Parameter	Value
Number of Nodes	20	Number of Rounds	3
Nodes Distribution	200 m × 200 m	Time per Round	90 time units
Number of Cluster Head	3	Number of Frames per Round	5
Initial Energy of Node	0.2 J	Sim-Time-Limit	200 s

**Table 2:** Parameter values of trust model.

Parameter	Value	Parameter	Value
MAX	100	${\mathrm{TH}}_{\mathrm{sp}}$	5
$\left({}^{ddt}K,{}^{rrt}K\right)$	(0.2, 0.5)	$\left(\lambda_{1},\lambda_{2}\right)$	(0.4, 0.6)
(α,β,γ)	(0.2, 0.3, 0.5)	$\left({\mathrm{Th}}_{\mathrm{abn}}^{\mathrm{behavior}},{\mathrm{Th}}_{\mathrm{susp}}^{\mathrm{behavior}}\right)$	(70, 80)
$\left({\mathrm{Th}}_{\mathrm{abn}}^{\mathrm{data}}{,\mathrm{Th}}_{\mathrm{susp}}^{\mathrm{data}}\right)$	(70, 80)	$\tau_{\mathrm{behavior}}$	1.1
$\tau_{data}$	1.1	$\left(\phi_{1},\phi_{2},\phi_{3}\right)$	(0.3, 0.3, 0.4)
$\left(\varepsilon_{1},\varepsilon_{2}\right)$	(0.5, 0.5)	$\left({\mathrm{Th}}_{\mathrm{abn}}{,\mathrm{Th}}_{\mathrm{susp}}\right)$	(70, 80)
δ	0, 1, 2	-	-

**Table 3:** Cluster heads and nodes within a cluster in each round.

Round	Cluster Heads (Index)	Node in the Cluster (Index)
1	1	7, 9, 13, 16
	2	0, 3, 5, 6, 11, 12, 14, 17
	8	4, 10, 15, 18, 19
2	0	3, 5, 6, 9, 11, 12, 17
	1	13, 16
	15	7, 8, 10, 18, 19
3	1	8, 9, 10, 11, 13, 16, 18
	6	7, 17
	19	15
